# A Half-Day Genome Sequencing Protocol for Middle East Respiratory Syndrome Coronavirus

**DOI:** 10.3389/fmicb.2021.602754

**Published:** 2021-02-19

**Authors:** Kwan Woo Kim, Sungmi Choi, Su-Kyoung Shin, Imchang Lee, Keun Bon Ku, Seong Jun Kim, Seil Kim, Hana Yi

**Affiliations:** ^1^Interdisciplinary Program in Precision Public Health, Korea University, Seoul, South Korea; ^2^Institute for Biomaterials, Korea University, Seoul, South Korea; ^3^Korea Convergent Research Center for Emerging Virus Infection, Korea Research Institute of Chemical Technology, Daejeon, South Korea; ^4^Microbiological Analysis Team, Group for Biometrology, Korea Research Institute of Standards and Science (KRISS), Daejeon, South Korea; ^5^Department of Bio-Analysis Science, University of Science and Technology, Daejeon, South Korea; ^6^School of Biosystems and Biomedical Sciences, Korea University, Seoul, South Korea

**Keywords:** MERS (Middle East respiratory syndrome), coronavirus, universal primer, whole-genome sequencing, MinION long-read sequencing

## Abstract

Recent coronavirus (CoV) outbreaks, including that of Middle East respiratory syndrome (MERS), have presented a threat to public health worldwide. A primary concern in these outbreaks is the extent of mutations in the CoV, and the content of viral variation that can be determined only by whole genome sequencing (WGS). We aimed to develop a time efficient WGS protocol, using universal primers spanning the entire MERS-CoV genome. MERS and synthetic Neoromicia capensis bat CoV genomes were successfully amplified using our developed PCR primer set and sequenced with MinION. All experimental and analytical processes took 6 h to complete and were also applied to synthetic animal serum samples, wherein the MERS-CoV genome sequence was completely recovered. Results showed that the complete genome of MERS-CoV and related variants could be directly obtained from clinical samples within half a day. Consequently, this method will contribute to rapid MERS diagnosis, particularly in future CoV epidemics.

## Introduction

Recent consecutive outbreaks of Betacoronavirus, including the severe acute respiratory syndrome (SARS), Middle East respiratory syndrome (MERS), and coronavirus disease (COVID-19), have severely impacted the human population. To date, seven types of coronaviruses (CoVs) have caused epidemics in the world, of which four are mild types (229E, NL63, OC43, and HKU1), while three are severe types (SARS-CoV-1, SARS-CoV-2, and MERS-CoV). Among them, MERS-CoV first appeared in Saudi Arabia in 2012 following zoonotic transmission from camels to humans (Zaki et al., 2012) and subsequently spread to 27 countries across all continents, resulting in 2494 laboratory-confirmed cases of infections with a case–fatality rate of 34.4% (May 2020, WHO) (World Health Organization ([WHO], 2019).

For MERS-CoV diagnosis, WHO currently recommends real-time reverse transcription polymerase chain reaction (rRT-PCR) targeting the following three genes: upstream of the E protein gene (upE), open reading frame 1b (ORF 1b), and open reading frame 1a (ORF 1a). The nucleocapsid (N) protein gene can be further analyzed to enhance the sensitivity. The rRT-PCR method is highly sensitive for detecting known MERS-CoV, but it cannot determine the extent of genomic mutations. Therefore, for variant detection, WHO recommends phylogenetic analyses using a complete genome sequence (World Health Organization [WHO], 2018). The whole genome sequencing (WGS) method is not only beneficial for mutant detection but also for tracing the origin of the virus and route of transmission, consequently establishing prevention strategies (Gilchrist et al., 2015).

Since the amount of viral nucleic acid in clinical samples is insufficient for direct genome sequencing, a precedent nucleic acid amplification step is required. However, constructing universal primers that effectively span the entire genome is difficult because viral genomes within the same species can greatly vary with a high mutation rate. Therefore, strain-specific primers have been designed for each viral strain, with the expenditure of time and effort. However, recently, universal primer sets have been successfully developed by a few leading research groups. For example, the conserved primer sets for each protein region of influenza virus have been developed for quick genome sequencing (Wang et al., 2015). Additionally, universal primer sets have been developed for the HIV-1 genome to locate the mutation sites that are responsible for drug resistance (Gall et al., 2012). These reports encouraged us to develop universal primers for MERS-CoV in the present study. To date, various methods have been used to amplify the MERS-CoV genome (Van Boheemen et al., 2012; Kim et al., 2015; Moreno et al., 2017; Yusof et al., 2017); however, universal primers appropriate for lineage C of Betacoronavirus, which MERS-CoV belongs to, are yet to be developed.

Despite the usefulness of the full-length genome, a major disadvantage is that genome amplification and sequencing is time consuming. It has therefore been impossible for the WGS method to primarily be used as an on-site diagnostic method. To significantly reduce the testing time, universal primers for MERS-CoV lineage C are needed, in addition to the establishment of a more efficient sequencing protocol. Accordingly, in the present study, we developed a WGS protocol that sequences the viral genome within half a day.

## Materials and Methods

### Preparation of MERS-CoV/KOR/KNIH/002_05_2015 RNA

The patient-derived MERS-CoV strain KNIH/002_05_2015 was obtained from Korea National Institute of Health (KNIH) and inoculated into cultured Vero E6 cells. The virus cultivation and RNA extraction were performed in a biosafety level-3 (BSL-3) laboratory at the Korea Research Institute of Chemical Technology (KRICT), as previously described.

### Synthesis of NeoCoV Genomic DNA

To evaluate the universality of the developed primers, Coronavirus Neoromicia/PML-PHE1/RSA/2011 (NCBI accession no. KC869678) was chosen as an experimental strain because it belongs to the same family as MERS-CoV (Hashemi-Shahraki et al., 2013). Eleven genomic fragments of 2940 bp were synthesized using a commercial service (GENEWIZ Inc.). Thereafter, to allow the designed forward and reverse primer pair to localize in a single genomic segmental DNA, each two-consecutive-genome-fragment was ligated into a 6 kb long fragment extension as described previously (Messing and Vieira, 1982). The resulting 14 extended fragments were used as the template for PCR amplification.

### Primer Design and Experimental Screening

The genome sequences of MERS-CoV and related viruses were derived from Virus Pathogen Resources (ViPR) (Pickett et al., 2012). The derived genome sequences were aligned using ViPR sequence alignment function. Following the alignment, conserved regions were selected and the candidate primers were chosen based on the following criteria: amplicon length, 2.5–3 kb; primer length, 18–23 nt; Tm, 53–57°C; overlaps between amplicons >100 bp; number of degenerate bases <2; and number of inosine molecules <1.

### cDNA Synthesis

For the synthesis of first strand cDNA, Superscript III First-Strand Synthesis SuperMix (Cat No. 18080-400, Invitrogen) was used. The initial master mix contained 1 μl RNA (50 ng/μl or 10 ng/μl), 1 μl random hexamers (5′-NNNNNN-3′, 50 ng/μl), 1 μl annealing buffer, and 5 μl RNase/DNase-free water. The mixture was incubated at 65°C for 5 min, and thereafter kept on ice for 1 min. Following the reaction, 10 μl 2× First-Strand Reaction Mix and 2 μl SuperScript III/RNaseOUT Enzyme Mix were added to the master mix and further incubated at 25°C for 10 min, 55°C for 90 min, and 85°C for 5 min.

### PCR Amplification

The following reagents were used for PCR amplification: 2 μl cDNA, 1 μl forward primer (10 pmol/μl), 1 μl reverse primer (10 pmol/μl), 10 μl KAPA HotStart ReadyMix (Roche), and 6 μl distilled water. The mixture was initially heated at 98°C for 30 s, and next, the mixture was subjected to a cycle comprising denaturation (at 98°C for 5 s), annealing (at 55°C for 30 s), and extension (at 72°C for 2 min), which was repeated 30 times, and finally further elongation at 72°C for 2 min. The PCR product was then purified using QIAquick^®^ PCR Purification Kit (Qiagen). The purified amplicons were quantified and pooled into a single mixture to construct sequencing libraries.

### Sequencing and Assembly

Purified amplicon mixture was sequenced using MinION (Oxford Nanopore), MiSeq (Illumina), and RS II (PacBio) according to the manufacturers’ instructions. The amount of input DNA for sequencing library construction was 1.0–1.5 μg, 130 ng, and 10 μg for each method, respectively.

The MinION sequencing library was constructed with Ligation Sequencing Kit (Oxford Nanopore) and then 1D sequencing was performed using R9.4 flow cells (Oxford Nanopore). A laptop with i7 CPU, 8 Gb RAM, and 128 GB solid state hard disk was used for MinION sequencing and base calling. The quality control and raw data processing were done using EPI2ME program (Oxford Nanopore). Base calling was performed with Q-score 7 using Guppy ver.3.4.4+a296acb (Oxford Nanopore).

The MiSeq sequencing library was constructed with Nextera XT DNA Library Preparation Kit (Illumina) and sequenced with the 300 bp paired-end protocol. The MinION and MiSeq data were assembled using CLC Genomic Workbench 9.3.5 (Qiagen).

The PacBio 10 kb sequencing library was constructed with SMRTbell Template Prep Kit 2.0 (PacBio) and sequenced with RS II. The raw data were filtered and aligned using SMRT Analysis program (PacBio) with Long Amplicon Analysis (LAA) option. The resulting long contigs were further manually cured using CodonCode aligner 8.0.2 (CodonCode Corporation).

The phylogenetic trees were constructed using the consensus sequence with MEGA program (Kumar et al., 2016).

### Preparation of Animal Serum Sample

To evaluate the clinical applicability of the developed method, the synthetic clinical sample was prepared by diluting the cultured viral particles present in mouse serum. Serum of a 16-week old female mouse was extracted and the experimental procedure was approved by KRICT (IACUC 2018-8A-09-01). Dilution series of the serum with three different viral concentrations (4.4 × 10^5^, 4.4 × 10^4^, and 4.4 × 10^3^ pfu/140 μl) were prepared. RNA was extracted from 140 μl of serum using the QIAamp viral RNA extraction Kit (Qiagen, Hilden, Germany) and eluted in 60 μl of elution buffer. Thus, the viral concentration in the extracted RNA was equivalent to 7.3 × 10^3^, 7.3 × 10^2^, and 7.3 × 10^1^ pfu/μl.

## Results

### Selection of Universal Primers for MERS-CoV

**FIGURE 1:**
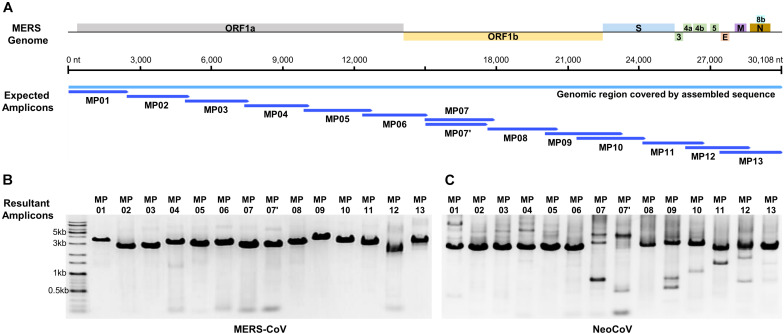
Graphical summary of the designed primer sets and the results of the genome amplification. **(A)** Localization of designed amplicons on the MERS-CoV genome. **(B)** PCR result of MERS-CoV genome amplification. **(C)** PCR result of NeoCoV genome amplification.

Based on the alignment results of the MERS-CoV-related genome, 68 primer candidates were obtained and subjected to experimental screening through reverse transcription and PCR. Consequently, 14 primer pairs that spanned the entire genome of MERS-CoV were obtained and named as the MP primer set (Figure 1A and Table 1). When we checked the primer specificity against the NCBI database using basic local alignment search tool (BLAST), the primers did not bind to any viruses other than MERS-CoV. The complete genome sequences of HCoV-OC43 (NC_006213), HCoV-HKU1 (NC_006577), SARS-CoV-1 (NC_004718), SARS-CoV-2 (NC_045512), HCoV-229E (NC_002645), and HCoV-NL63 (NC_005831) were downloaded and further evaluated *in silico* to confirm the specificity of the 14 primer pairs. The results demonstrated the Betacoronavirus lineage C specificity of the chosen primers. In addition, the primers did not bind to other locations in the genome, except the ones we identified in the current study. All the 14 primer pairs included in MP primer set exhibited target site specificity by forming a single band amplicon having the expected size (3 kb) (Figure 1B).

**TABLE 1:** Contents of the MP primer set developed for whole genome sequencing of MERS-CoV.

No.	Primer pair	Sequence (5′→ 3′)	Product Size (bp)
MP01	37F	CGTTCTCYTGMAGRACTTTG	2675
	2712R	GTAGGWGACTATGTYATTATTA	
MP02	2654F	GGTTGAAACTGTTGTNGGTC	2618
	5272R	GCATGGTTTGGAGAGAGTG	
MP03	5079F	CTTGATTTRTTGAAGGACATT	2677
	7756R	TAGATAAGTTGAAGTTCAAAGA	
MP04	7566F	GGGAATACCTTCATCTGTGA	2722
	10288R	TGAAGTTGACTGTCGATGT	
MP05	10056F	GTTGARGCTTGTATGGTTC	2871
	12927R	AATCTTAACAACCTTCATCG	
MP06	12513F	GCTGGGGCTTTGTGGGAC	2653
	15248R	CATCTTATGGGTTGGGATTAC	
MP07	15119F	CAGTACCATCAGAAAATGCT	2940
	18059R	CCTGCTTATGCACCAACA	
MP07′	15146F	ATGGCTGCAACTCGTGG	2596
	17741R	TGGAGTAAGGCAGTCTTTA	
MP08	17741F	TGGAGTAAGGCAGTCTTTA	2957
	20698R	ATCTATTCCTATGCCTCGC	
MP09	20154F	GTCGCTCTTGTAGTGACTT	3261
	23415R	TGTGTYAGTGTKCCTGTTTC	
MP10	21473F	TCYACTGATGTKCTTGTTAAC	2890
	24363R	ATTCCWTTTGCACAGAGTAT	
MP11	24228F	TGTGCTCARTATGTNGCTGG	2598
	26826R	GATTTTAACGAACTAYGGC	
MP12	26029F	GATGGAGGAATMCCYGAYG	2723
	28752R	GTACCTCTTAATGCCAATTC	
MP13	27464F	CGCGATTCAGTTCCTCTTC	2642
	30106R	CAAAWYATCTAATTAGCCTAAT	

### Optimization of RT and PCR Procedures

We synthesized cDNA using reverse-transcription to amplify the single-strand RNA CoV genome. To optimize cDNA priming method, the following two experimental methods were performed and evaluated. Method-A, which is adopted in this study, used a combination of a random hexamer (for RT) plus MP primers (for PCR). Method-B used MP primers both for RT as well as PCR (data not shown). When comparing the two methods, method-B showed a better detection limit for cDNA synthesis compared to method-A. However, in terms of specificity, method-A exhibited better amplicon quality with a single-band in gel electrophoresis, while method-B produced multi-bands in almost all primer pairs. Since a gel-elution step was required for such multi-band amplicons and it caused DNA loss and resulted in an unmet amount of DNA required for sequencing platforms, we decided to choose random hexamer priming for the reverse-transcription step.

In addition, we tried multiplex PCR to save on handling time and cost. The first trial used one reaction tube with whole 14 primer pairs, and the second trial used two tubes each containing odd-numbered or even-numbered primer pairs. The amplification resulted in multiple bands with unexpected sizes, but the PCR products were purified and processed to PacBio RS II sequencing. Due to the high amplification bias and unwanted amplicons, we failed to obtain a complete genome sequence from any of the multiplex PCR products. Thus, we did not consider multiplex PCR as the amplification method.

To determine the detection limit of the experiments, RNA concentrations of 1, 10, and 50 ng/μl were prepared and used for template RNA for cDNA synthesis. Once the PCR reaction was completed, the 10 and 50 ng/μl samples showed detectable bands in gel electrophoresis, but the 1 ng/μl sample did not. Thus, the minimum concentration and amount of template RNA required for cDNA synthesis was determined to be 10 ng/μl and 10 ng, respectively.

### Comparison of Sequencing Methods

To determine the optimal sequencing method, various sequencing techniques were evaluated. A consensus sequence of MERS-CoV strain KNIH/002_05_2015 was determined using the most frequent nucleotide found at each position in a sequence alignment of the three complete genome sequences derived from MinION, MiSeq, and RS II. The resultant single consensus sequence was deposited in GenBank (accession number MT387202). The consensus was used as a control sequence for comparison of the three genome sequence results from three different sequencing techniques. The MinION, MiSeq, and RS II sequences covered 99.81–99.91% regions of the entire MERS-CoV genome with a sequencing accuracy of 99.98–100%, confirming that applying any of these methods would yield a sequence that covers the entire genome using the MP primers (Table 2). With regard to the sequencer running time, MinION was the most time efficient technique, requiring only 5–30 min of sequencer running time and 30 min of assembly time. The 15-min MinION sequencing resulted in 0.5 Gb of raw data with 740.97× sequencing depth. Following sequence assembly, one long contig of full-length genome was obtained. This 15-min MinION assembled contig predominantly matched the consensus sequence, where only 17 out of 30108 bp were different, confirming the 99.9435% accuracy rate (Supplementary Figure 1). Among the 17 different nucleotide sequences, 13 were originated from homopolymeric errors (Rang et al., 2018) and four were from sequencing technical errors.

**TABLE 2:** Comparison of sequencing data depending on sequencing platforms.

Sequencer	Running time	Raw data size (no. of Reads)	Sequencing depth (average)	% Genome coverage* (contig length)	% Accuracy^#^ (no. of mismatch)
MinION	5 min	167 Mb (3,697)	228×	99.9070 (30,080 bp)	99.9534 (14 nt)
	15 min	532 Mb (11,030)	741×	99.9469 (30,092 bp)	99.9435 (17 nt)
	30 min	1.24 Gb (25,700)	1,801×	99.9469 (30,092 bp)	99.9501 (15 nt)
RSII	10 h	17.1 Gb (84,774)	460×	99.8903 (30,075 bp)	100 (0 nt)
MiSeq	48 h	5.11 Gb (8,100,458)	23,700×	99.8106 (30,051 bp)	100 (0 nt)

*Genome coverage means the percentage area coverage of a reference (consensus sequence, 30,108 bp) by the assembled contig. ^#^Accuracy means the proportion of matched nucleotides in the assembled contig compared to a reference (consensus sequence, 30,108 bp).

### Validation of MP Primer Universality

When MP primers were applied to the NeoCoV gene fragment extension (6 kb) template, all amplifications proved to be successful (Figure 1C). This proved that MP primers can be applied to a neighboring clade with a genome sequence similarity as low as 85%, confirming the universality of these primers. Although several primer pairs (MP7, 7′, 9, 11, and 12) presented multi-bands in NeoCoV amplification unlike in MERS-CoV, the sequencing was successfully performed without any gel-elution step. The amplicon mixture of NeoCoV synthetic genomic DNA was sequenced by MinION and a single contig of the complete genome sequence was recovered. The result demonstrated the universality of the MP primer and the optimized experimental and analytical procedures developed in the present study.

### Animal Sample Applicability

When synthetic animal serum was evaluated, target amplicons were produced even at the lowest viral concentration (7.3 × 10^1^ pfu/μl). The amplicon mixtures from the lowest viral concentration were successfully sequenced using MinION and assembled into a complete genome sequence with an accuracy >99.00%. Considering the amount of input RNA (1 μl) required for reverse transcription reaction, having more than 73 viral particles would facilitate application of the experimental protocol developed in this study. We therefore decided to use 10^2^ viral particles per reaction as the detection limit of this newly developed method.

### Expected Coverage of MP Primers Within the Betacoronavirus Clade C

**FIGURE 2:**
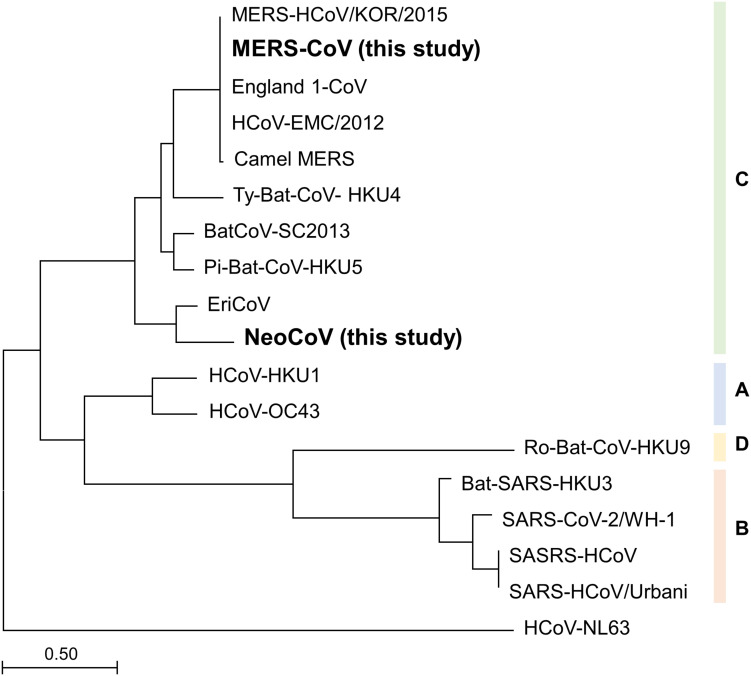
Maximum-likelihood tree based on spike protein subunit1 (S1) amino acid sequences. The farthest evolutionary distance between MERS-CoV and NeoCoV within the Betacoronavirus clade C demonstrates the universality of the MP primer set.

To infer the universality of MP primers *in silico*, phylogenetic analysis was performed using all the known species of Betacoronavirus clade C. Maximum likelihood tree analysis of spike protein subunit1 (S1) amino acid sequence showed that MERS-CoV and NeoCoV had the farthest evolutionary distance among the six known species within the Betacoronavirus clade C (Figure 2). Therefore, according to the evolutionary trend of S1 proteins, the two species analyzed in this study (MERS-CoV and NeoCoV) widely cover the diversity of members belonging to the Betacoronavirus clade C. However, based on spike protein subunit2 (S2) proteins, NeoCoV was recovered as a sister group of MERS-CoV, unlike the S1 tree (Supplementary Figure 2).

## Discussion

The designed MP primer set that spanned the entire genome of MERS-CoV was also successfully applied to NeoCoV, thus verifying its universality within the Betacoronavirus clade C. Despite the large genome sequence difference (89.6%) between the two viral species, MP primers worked well for WGS of both, indicating that the primers can universally be applied to clade C of Betacoronavirus. In addition, the MP primer set was able to amplify and recover the MERS-CoV genome contained in animal serum, suggesting that the developed experimental procedure can also be applied to clinical samples. Another major advantage of this MP primer set was that it covered nearly the complete genome sequence of the tested CoVs, despite their large genome size (30 kb).

**FIGURE 3:**
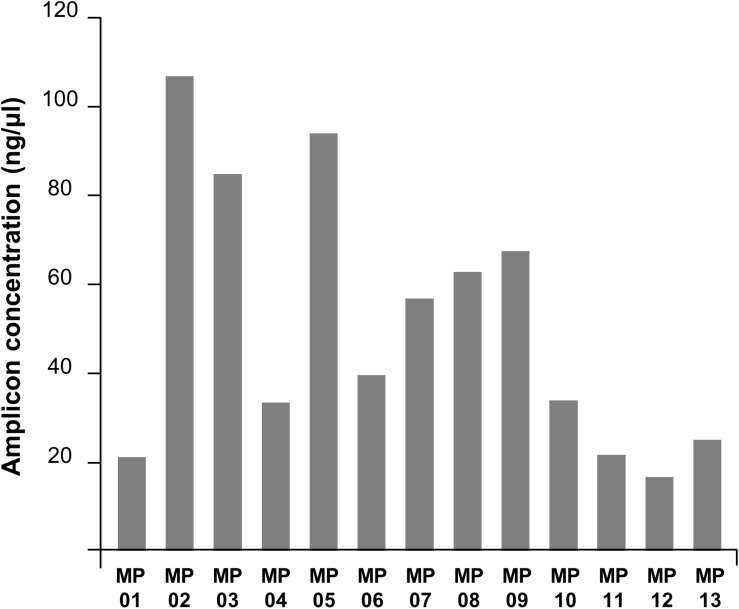
PCR amplification efficiencies of MP primers. The DNA concentration in the PCR product varied depending on primers showing the amplification efficiency bias across the MERS-CoV genome region.

Amplification bias was found among target locations within the genome (Figure 3). The primer number 02 was the most efficient region and number 12 was the least. Overall, the amplification efficiency was higher in the anterior part of the genome compared to the posterior region, potentially due to RNA degradation. RNA degrades for numerous reasons, but it mostly occurs from the 3′-end, a protein coding region where messenger RNA is present (Van Hoof and Parker, 2002). This affected the 21–30 kb region, where the main protein coding genes of MERS-CoV are located, and this could have resulted in the differences observed in reverse-transcription efficiency. Secondly, the efficiency of transcription and amplification of a specific region in the gene was significantly reduced due to yet unknown reasons. The forward primer of MP7 is located in the RdRp gene, and the reverse primer is in the ORF 1b gene. Previously, WHO reported that the PCR efficiency of the RdRp and OFR 1b regions are lower than that of other regions; this was consistent with the fact that the amplification efficiency of MP7 was low in our experiment. Similarly, nucleic acid amplification test applied to hCoV-EMC reported that the sensitivity for the ORF 1b assay was lower than that for upE (Corman et al., 2012a). Additionally, other studies demonstrated that the sensitivity of RdRp was lower than that of N (Corman et al., 2012b), and ORF 1a was shown to have the highest sensitivity among all the assays (Corman et al., 2012a,b). SARS-CoV-2, which is currently causing a pandemic, has also been reported to have higher amplification efficiency in ORF 1a than in ORF 1b (Jung et al., 2020). Based on the previous reports, we decided that the MP7 region was difficult to cover with a single primer, and thus, we added a supplementary primer named MP7′ to enhance the universality of that specific region.

Real-time reverse transcription polymerase chain reaction is a useful method in terms of response-time (<2 h) and sensitivity, but sequence variations are not easily detected because the assay targets a very short sequence (100 bp). In reality, the regions where mutations of MERS-CoV frequently occur are spike glycoprotein (Kim et al., 2016) and S1 (Anthony et al., 2017), but the rRT-PCR cannot provide any information for variant detection. WGS is an irreplaceable method to obtain precise information on viral mutation, but we have to overcome the lengthier sequencing time and accuracy issues. Recently, the MinION-based rapid genome sequencing directly from clinical samples was reported for Ebola (Quick et al., 2016) and Zika (Quick et al., 2017) viruses. Based on their success, we further optimized the MinION process to suit MERS-CoV. Consequently, the sequencing and analysis of the entire MERS-CoV genome were completed in 1 h with a sequencing accuracy of 99.9435%.

The consensus sequence determined in this study (MT387202) showed 8 nt variation compared to the reference genome sequence of patient-derived MERS-CoV strain KNIH/002_05_2015 (KT029139). The variation was observed on orf1a, orf1b, orf4a, orf4b, and spike glycoprotein. Those level of SNPs could be cell-culture adaptive mutations, which are frequently observed when putting patient-originated viruses in to culturing conditions for a long time (Dargan et al., 2010; Oka et al., 2014). According to the phylogenetic trees inferred using the S1 and S2 domain of spike protein independently, a faster evolution rate was observed in S1 than S2 in the test strain in accordance with other corona viruses (Anthony et al., 2017; Sohrab and Azhar, 2020).

We wanted to verify the applicability of the developed protocol to clinical samples, but we could not recruit any patients since the MERS-CoV epidemic has already passed. To overcome the lack of clinical samples, synthetic animal serum samples were used instead. As previously reported elsewhere, MERS-CoV and SARS-CoV-2 were detected in patient serum samples using nucleic acid amplification methods (Oh et al., 2016; Zheng et al., 2020). Thus, we chose serum as the diluent of viral particles. Considering that the viral load in MERS patients is 103–109 copy/ml (Oh et al., 2016), because the detection limit in animal serum samples was 100 viral particles per reaction, the newly developed method seems to also be suitable for clinical samples.

Given the practical working time (6 h), acceptable sequencing error rate, and portability of the sequencer, we anticipate that our developed protocol can become a powerful tool for studying emerging variants of MERS-CoV in the field during future outbreaks.

## Data Availability Statement

The original contributions presented in the study are publicly available. This data can be found here in GenBank under accession number MT387202.

## Ethics Statement

The animal study was reviewed and approved by Korea Research Institute of Chemical Technology. Written informed consent was obtained from the owners for the participation of their animals in this study.

## Author Contributions

KWK, SC, S-KS, and IL performed the experiments and conducted the bioinformatics analyses for sequencing data. SK and HY designed the study and interpreted the data. KBK and SJK cultivated the viruses and performed the animal experiments. KWK, SC, SK, and HY were the major contributors in writing the manuscript. All authors read and approved the final manuscript.

## Conflict of Interest

The authors declare that the research was conducted in the absence of any commercial or financial relationships that could be construed as a potential conflict of interest.

## Supplementary Material

Supplementary Figure 1 Comparison of sequence variation among sequencing running time on MinION platform. The MERS-CoV genome sequence determined according to each MinION sequencing running time was compared against the consensus sequence derived from three sequencing platforms. The nucleotide positions with sequence heterogeneity are marked in colored blocks. Dashes indicate gaps in the sequence of one genome relative to its counterpart.

Supplementary Figure 2 Maximum-likelihood tree based on spike protein subunit2 (S2) amino acid sequences. The MERS-CoV and NeoCoV viruses belonging to the Betacoronavirus clade C demonstrates the level of universality of the MP primer set.
