# Supplementary figures and images for: A Half-Day Genome Sequencing Protocol for Middle East Respiratory Syndrome Coronavirus
Source: Front Microbiol. 2021 Feb 19;12:602754. doi: 10.3389/fmicb.2021.602754 (PMC7933487; doi:10.3389/fmicb.2021.602754)

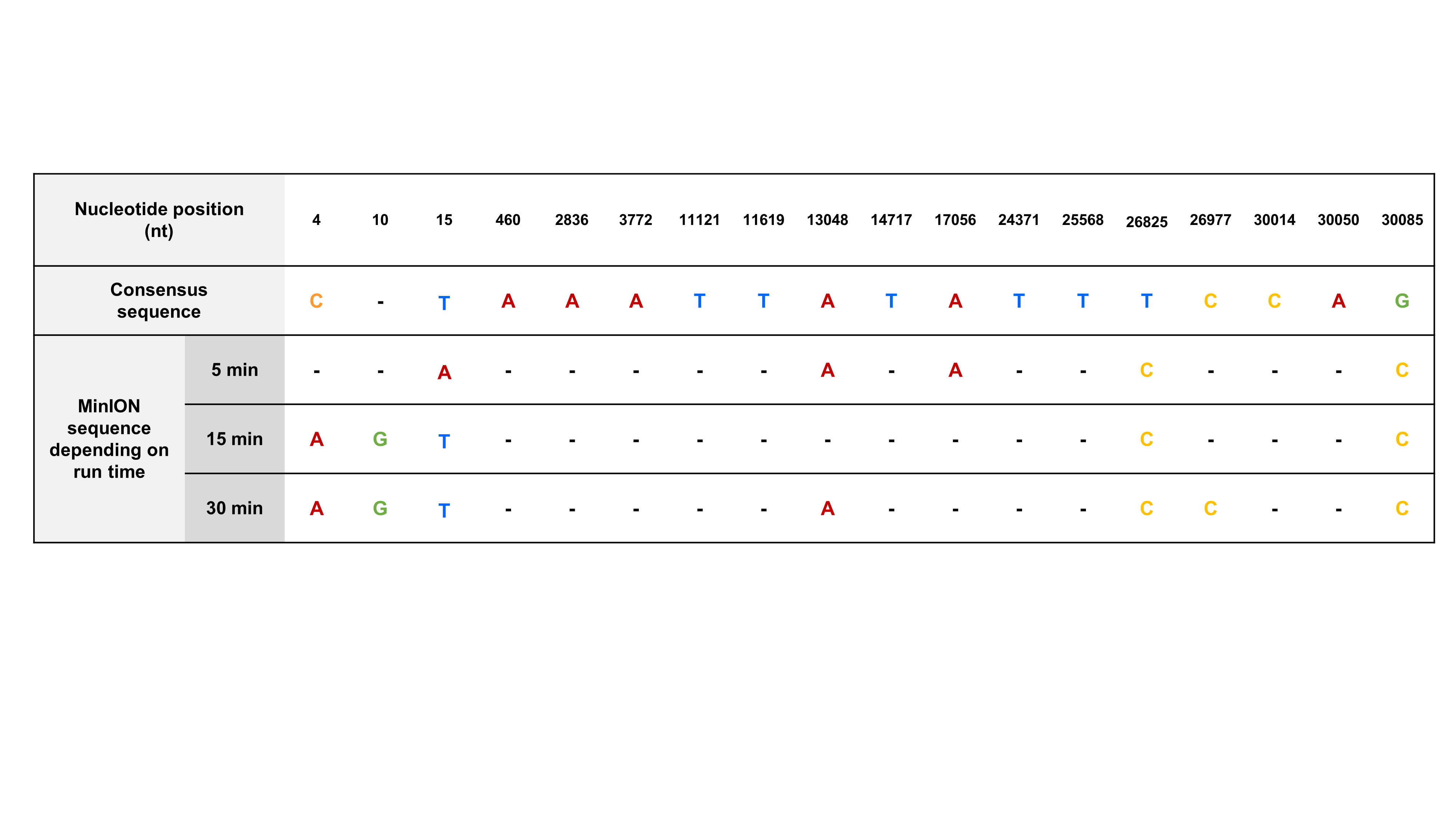

Supplement: Supplementary Figure 1 — Comparison of sequence variation among sequencing running time on MinION platform. The MERS-CoV genome sequence determined according to each MinION sequencing running time was compared against the consensus sequence derived from three sequencing platforms. The nucleotide positions with sequence heterogeneity are marked in colored blocks. Dashes indicate gaps in the sequence of one genome relative to its counterpart. [file Image_1.tif]

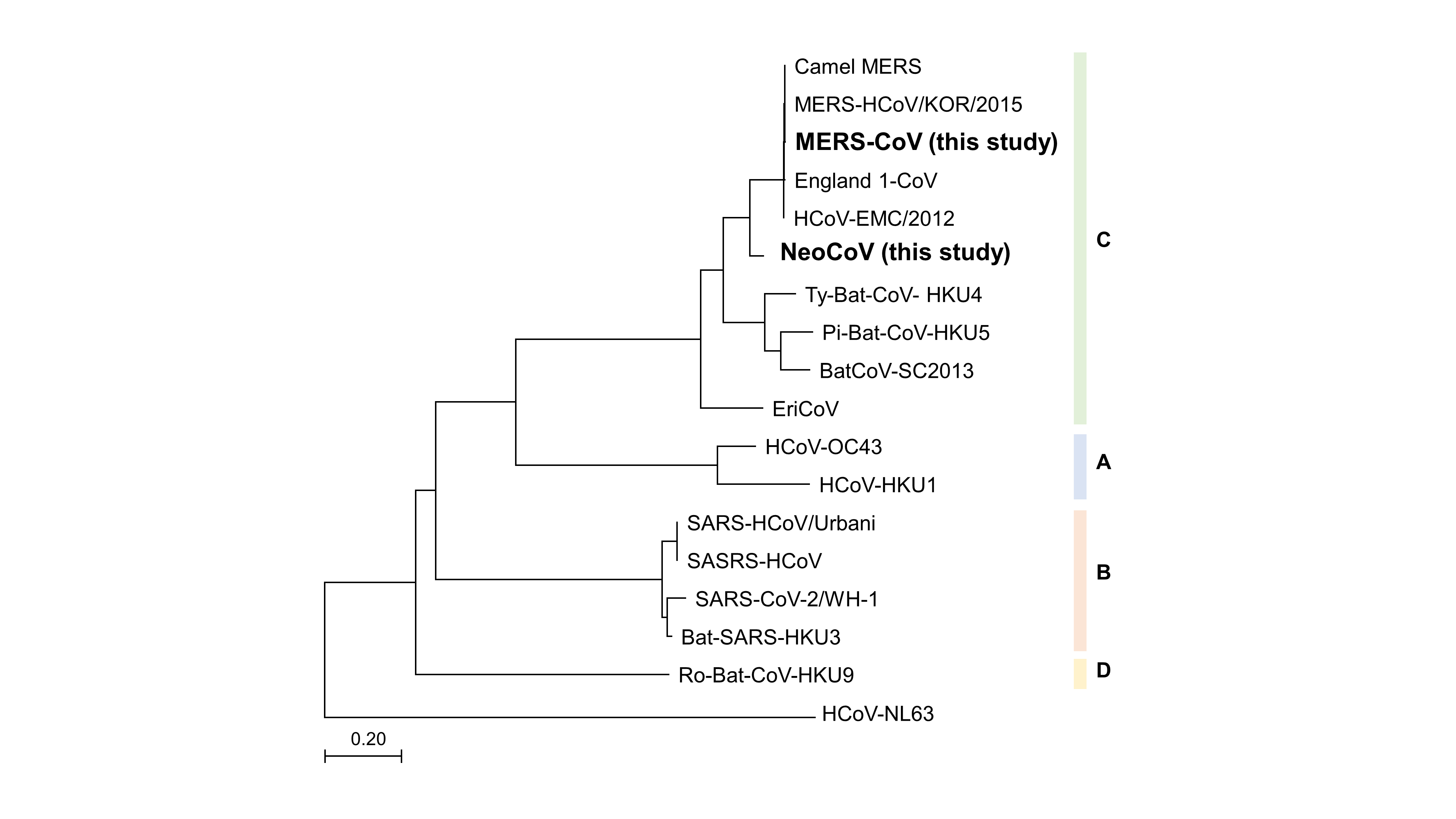

Supplement: Supplementary Figure 2 — Maximum-likelihood tree based on spike protein subunit2 (S2) amino acid sequences. The MERS-CoV and NeoCoV viruses belonging to the Betacoronavirus clade C demonstrates the level of universality of the MP primer set. [file Image_2.tif]
